# Robotic-assisted needle placement in CT-guided percutaneous ablation in the abdomen: the SaFE post-market study

**DOI:** 10.1007/s11547-026-02181-5

**Published:** 2026-02-20

**Authors:** Baptiste Bonnet, Lambros Tselikas, Paul Beunon, Arnaud Pouvelle, Eloi Varin, Alice Boilève, Genti Xhepa, Thierry De Baère, Frédéric Deschamps

**Affiliations:** 1 Service d’Imagerie Thérapeutique, Département d’Anesthésie, Chirurgie et Interventionnel (DACI), Gustave Roussy, 114 Rue Édouard Vaillant, 94805 Villejuif, France; 2 Centre d’Investigation Clinique BIOTHERIS, INSERM CIC1428, Gustave Roussy, Villejuif, France; 3 Faculté de Médecine, Paris-Saclay Université, 94276 Le Kremlin-Bicêtre, France; 4 Service d’Oncologie Digestive, Département d’Oncologie Médicale, Gustave Roussy, 94805 Villejuif, France

**Keywords:** Tomography, X-ray computed procedure, Ablation techniques, Abdomen, Neoplasm, Robotics

## Abstract

**Purpose:**

To assess the feasibility, safety, and accuracy of robotic needle placement for abdominal percutaneous interventions in a real-world, post-market setting.

**Patients and methods:**

This prospective single-center study assessed a robotic guidance device for CT-guided needle placement during abdominal thermal ablation procedures. The primary endpoint was technical success, defined as successful robotic needle placement without technical failure (full manual insertion or two failed robotic attempts). Secondary endpoints included procedural safety, needle placement accuracy (3D deviation and manual adjustments categorized as minor [depth-only], moderate [lateral], and major [complete needle retrieval]), immediate ablation success, 2-month and 1-year local recurrence, and operator satisfaction (5-point Likert scale).

**Results:**

Between April 2022 and January 2023, 54 patients (one duplicate inclusion excluded) were analyzed (30 men, 24 women); mean age 64.7 (± 12.9). Most had metastatic disease (74.1%). Target organs included mainly the liver (68.5%) and the kidney (24.1%). Mean lesion diameter was 24.7 mm (± 13.1), with 59.0% considered technically challenging. Ablation modalities included microwave (63.3%), cryoablation (35.0%), and radiofrequency (1.7%). A total of 108 needles were placed (mean 1.8/patient), yielding a technical success rate of 94.4%. The mean final 3D accuracy after adjustments when required was 2.5 mm (± 3.7). Immediate ablation success was achieved in 98.4% lesions (60/61), with a mean minimal margin of 5.6 mm (± 3.2). Local recurrence occurred in 8.3% of cases at 2 months and in 25.6% at 1 year. Operator satisfaction averaged 3.1/4.

**Conclusion:**

Robotic guidance for CT-guided abdominal thermal ablation is feasible, safe, and provides high needle placement accuracy. Early oncologic outcomes appear comparable to conventional freehand techniques, supporting the integration of robotic systems into routine interventional radiology practice.

**Graphical Abstract:**

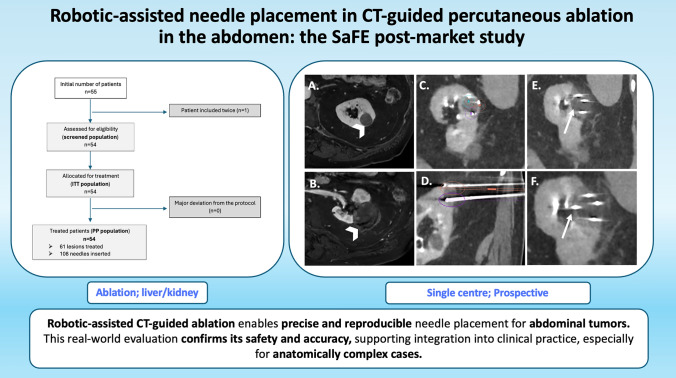

**Supplementary Information:**

The online version contains supplementary material available at 10.1007/s11547-026-02181-5.

## Introduction

The growing volume and complexity of interventional radiology procedures have driven substantial innovations to improve precision and clinical outcomes. Thermal ablation has emerged as a key minimally invasive therapeutic option, particularly for abdominal tumors, and is now integrated into major international guidelines [1–4]. Recent trials have further established its role, particularly for hepatic tumors, most notably the COLLISION trial, which supported thermal ablation for patients with small (< 3 cm) colorectal liver metastases as an alternative to surgery [5, 6]. Large-scale studies and meta-analyses have demonstrated that percutaneous ablation in renal oncology offers oncological outcomes comparable to partial or radical nephrectomy, with fewer complications and shorter hospital stays [7, 8].

As interventional radiology indications broaden, procedural standardization is becoming a clinical priority. Essential steps now include CT-guided needle placement verification and systematic post-ablation margin assessment, with correlation to oncologic outcome, as demonstrated in recent studies [9, 10] and under investigation in ongoing trials such as the ACCLAIM trial (Ablation with Confirmation of Colorectal Liver Metastases, NCT05265169).

In this context, robotic systems are gaining prominence as tools to optimize needle guidance and enhance reproducibility. They are designed to improve targeting precision, reduce radiation exposure, shorten procedure times, and minimize inter-operator variability [11–14]. Currently available systems range from simple navigation assistance platforms to fully integrated solutions that combine trajectory planning, robotic guidance, and ablation confirmation within a single workflow [15, 16].

The Epione® robotic system (Quantum Surgical, Montpellier, France) has recently been integrated into routine clinical practice following promising preclinical and early clinical evaluations [17–19]. A first-in-human study reported a technical success exceeding 95%, with no procedure-related complications [20], and subsequent retrospective analysis of 41 cases confirmed these findings [17].

This study reports the first prospective post-market clinical evaluation of this system for robotic-assisted abdominal CT-guided percutaneous thermal ablation, focusing on feasibility, safety, and accuracy under real-world conditions.

## Patients and methods

### Study design and objectives

This prospective post-market study was approved by the Institutional Review Board (no. 2022-107) and conducted in compliance with the national data protection authority regulations (CNIL MR-004; ID: 2224848). While written informed consent was waived under applicable regulations, all eligible patients were provided with non-opposition forms prior to their inclusion.

This study evaluated the feasibility, safety, and accuracy of robotic-assisted needle placement using the robotic system for CT-guided percutaneous thermal ablation of abdominal tumors. The first 20 patients had been previously included in a published retrospective analysis [17].

The primary endpoint was feasibility, defined by the technical success of robotic-assisted needle insertion. Technical failure was defined as requiring full manual insertion or unsuccessful insertion after two failed robotic attempts.

Secondary endpoints included safety, accuracy, immediate ablation success, local recurrence, and the operator’s satisfaction. All adverse events, severe or not, related to the device or to the procedure, were collected according to the Society of Interventional Radiology (SIR) classification [21]. Accuracy was measured using the 3D deviation (distance from the inserted needle tip to the planned trajectory tip, mm) as well as lateral (distance from inserted needle tip to its orthogonal projection on the planned trajectory, mm) and depth (distance between the orthogonal projection of the needle tip on the planned trajectory, and the planned trajectory tip, mm) specific deviations. Frequency and type of manual adjustments were recorded for each insertion, classified as minor (depth-only, forward or backward movement of the needle along the needle axis), lateral (correction of the needle trajectory axis), or major (complete withdrawal of the needle and full manual reinsertion). The immediate ablation success (yes/no) was evaluated by the radiologist and defined as complete tumor coverage with appropriate margins on immediate post-ablation enhanced CT, and the minimal margin obtained was measured (mm). Local recurrence was evaluated on imaging follow-up at 2 and 12 months. All nodular formations showing contrast enhancement and/or hypersignal diffusion on the edge of the ablation zone were considered as a recurrence. Lastly, the operator’s global satisfaction was evaluated at the end of the procedure using a 5-point Likert scale (0 = very dissatisfied, 4 = highly satisfied). Lesions were considered challenging if located in the hepatic dome, near the liver hilum (< 1 cm), adjacent to the liver capsule (< 1 cm), or near the renal pelvis (< 1 cm).

### Patient population

From April 2022 to January 2023, all consecutive adult patients undergoing robotic-assisted CT-guided percutaneous thermal ablation of abdominal tumor lesions using the robotic device were prospectively enrolled. The decision to proceed with CT-guided robotic ablation was made within a multidisciplinary tumor board. Exclusion criteria included contraindication to general anesthesia and pregnant or breastfeeding women.

### Robotic-assisted procedure

Procedures were performed using the robotic system, following the workflow described in previous reports [17, 20]. Briefly, all procedures were conducted under general anesthesia, with breath-hold reproducible apnea or jet ventilation [22] during imaging and needle insertion. To ensure spatial accuracy throughout the procedure and compensate for patient motion, a patient reference marker was attached to the patient’s skin and continuously tracked by the system. Needle trajectory planning was performed by the interventional radiologist using the initial CT scan within the integrated planning software, defining both the skin entry point and the target location. Once the trajectory(ies) was(were) finalized, the robotic arm was sent to the requested position along the planned axis, enabling the physician to insert the needle through the robotic needle guide, from the skin surface to the targeted lesion, in a single controlled motion. When multiple needles were required, all treatment probes were inserted sequentially using robotic assistance. For ancillary maneuvers or protective techniques (e.g., hydrodissection), needles were inserted using a conventional freehand approach. A control CT scan was subsequently performed to verify needle positioning. Manual adjustments were performed under CT- or ultrasound-guidance, if needed, to ensure optimal needle positioning for treatment, at the operator’s discretion. The selected ablation modality—microwave ablation, cryoablation, or radiofrequency ablation—was then applied according to the lesion characteristics, following the manufacturer’s guidelines.

To further illustrate the procedural workflow, two supplementary videos are provided: one demonstrating the trajectory planning process within the integrated software (Supplementary Video 1) and one illustrating robotic-assisted needle insertion during a clinical procedure (Supplementary Video 2).

All procedures were performed by one of six interventional radiologists (BB, LT, AP, EV, TDB, FD) with 3 to > 20 years of experience in freehand percutaneous thermal ablation procedures. Before the study, all operators had undergone training on the use of the robotic device.

### Statistical analysis

Statistical analyses were conducted by Delta Consultants (Eybens, France) using SAS® software version 9.4 (Cary, NC, USA). Quantitative variables were expressed as means and standard deviations, and categorical variables as frequencies and percentages. Analyses were performed at the patient, lesion, or needle level, as appropriate. Exploratory subgroup analyses examined needle placement accuracy according to the treated organ (liver or kidney), ablation modality (microwave or cryoablation), lesion complexity (challenging or non-challenging), and operator experience (< 5 or ≥ 5 years). Recurrence and 12-month progression-free survival (PFS) were also analyzed according to organ, ablation type, and lesion size (≤ 3 cm or > 3 cm). Comparisons of 12-month PFS used odds ratios (ORs) as an approximation of hazard ratios, derived from dichotomous outcomes (progression vs. no progression) at 12 months, with ORs, 95% confidence intervals, and *p*-values calculated using Fisher’s exact test. Continuous variables such as 3D deviation were compared using Welch’s *t*-test for independent samples, with a significance level set at *p* < 0.005.

## Results

### Study population

**Fig. 1:**
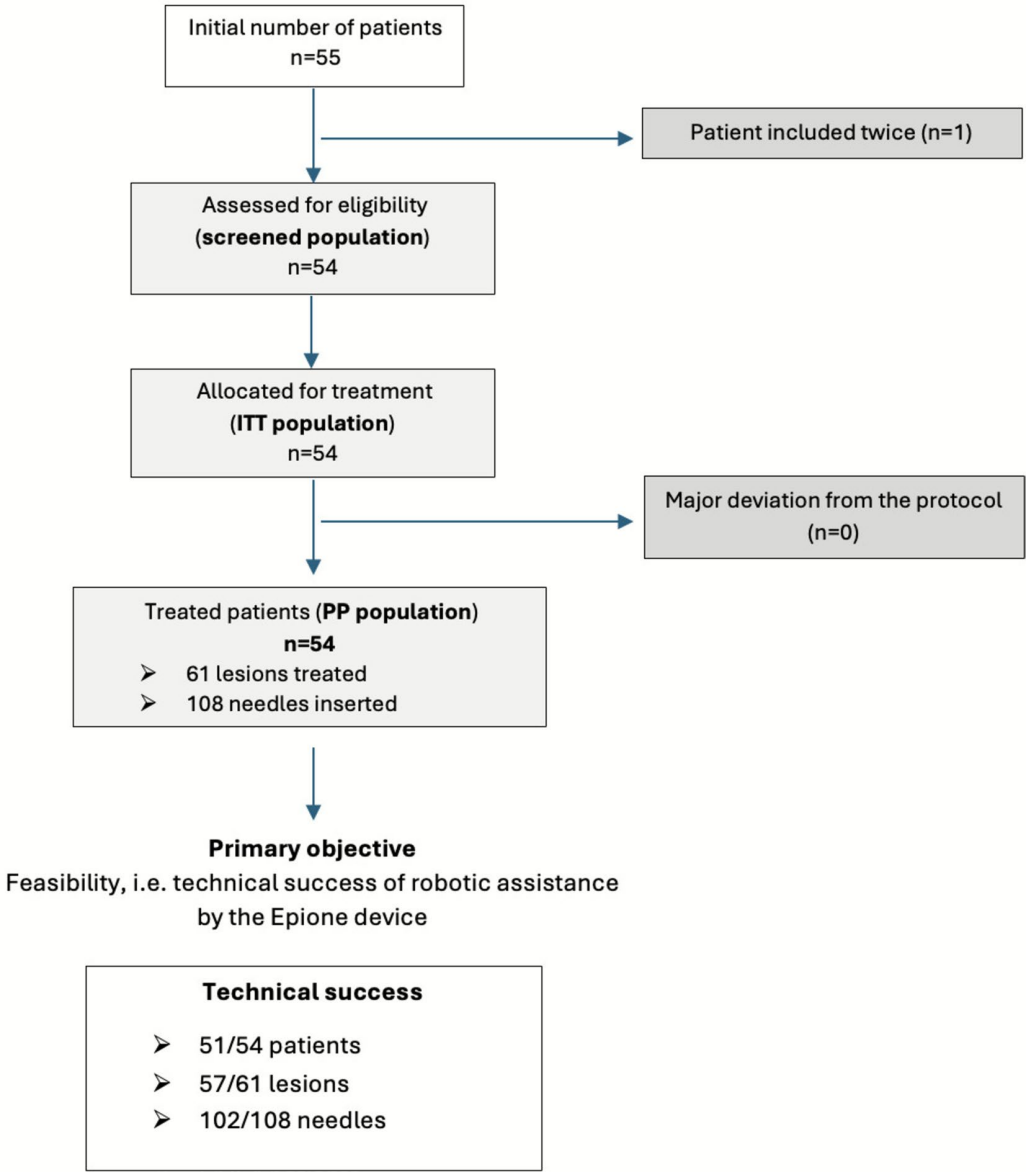
Study flowchart

Between April 2022 and January 2023, 55 patients underwent CT-guided thermal ablation with robotic assistance for abdominal lesions. Of these, 54 patients were included in the final analysis, with one patient excluded due to duplicate inclusion (Fig. 1). The cohort included 30 men (55.6%) and 24 women (44.4%), with a mean age of 64.7 years (± 12.9). Sixty-one lesions were targeted, with a mean size of 24.7 mm (± 13.1). Of these, 36 (59.0%) were classified as challenging based on their anatomical location. Lesions were treated with microwave ablation (*n* = 38, 63.3%), cryoablation (*n* = 21, 35.0%), or radiofrequency ablation (*n* = 1, 1.7%). Patients’ characteristics are summarized in Table 1.

**Table 1:** Patients’ lesion and procedure characteristics

Variables, *n* (%)	
*Patient data (n* = *54)*	
Gender	
Men	30 (55.6)
Women	24 (44.4)
Age, years, mean (± SD)	64.7 (± 12.9)
Organ treated	
Liver	37 (68.5)
Kidney	13 (24.1)
Adrenal gland	3 (5.6)
Retroperitoneum	1 (1.9)
Metastatic status	40 (74.1)
Primary tumor, if metastatic	
Colorectal	20 (50.0)
Neuroendocrine	7 (17.5)
Lung	4 (10.0)
Kidney	3 (7.5)
Others	6 (15)
Primary tumor, if not metastatic	
Liver	3 (21.4)
Kidney	11 (78.6)
*Lesion data (n* = *61)*	
Number of lesions per patient, mean (± SD)	1.3 (± 0.3)
Lesion size, mm, mean (± SD)	24.7 (± 13.1)
Treatment	
MWA	38 (63.3)
Cryoablation	21 (35.0)
RFA	1 (1.7)
Number of needles per lesion, mean [range]	1.8 [1–6]
Challenging lesions	36 (59.0)
*Procedure data (n* = *108 needles)*	
Number of needles per patient, mean (± SD)	1.8 (± 1.2)
Out-of-axial-plane trajectories	37 (34.3)
Duration of use of robotic device (from beginning of trajectory planning to end of needle insertion), min, mean (± SD)	26.8 (± 21.2)

*SD* standard deviation; *MWA* microwave ablation; *RFA* radiofrequency ablation

### Feasibility

**Fig. 2:**
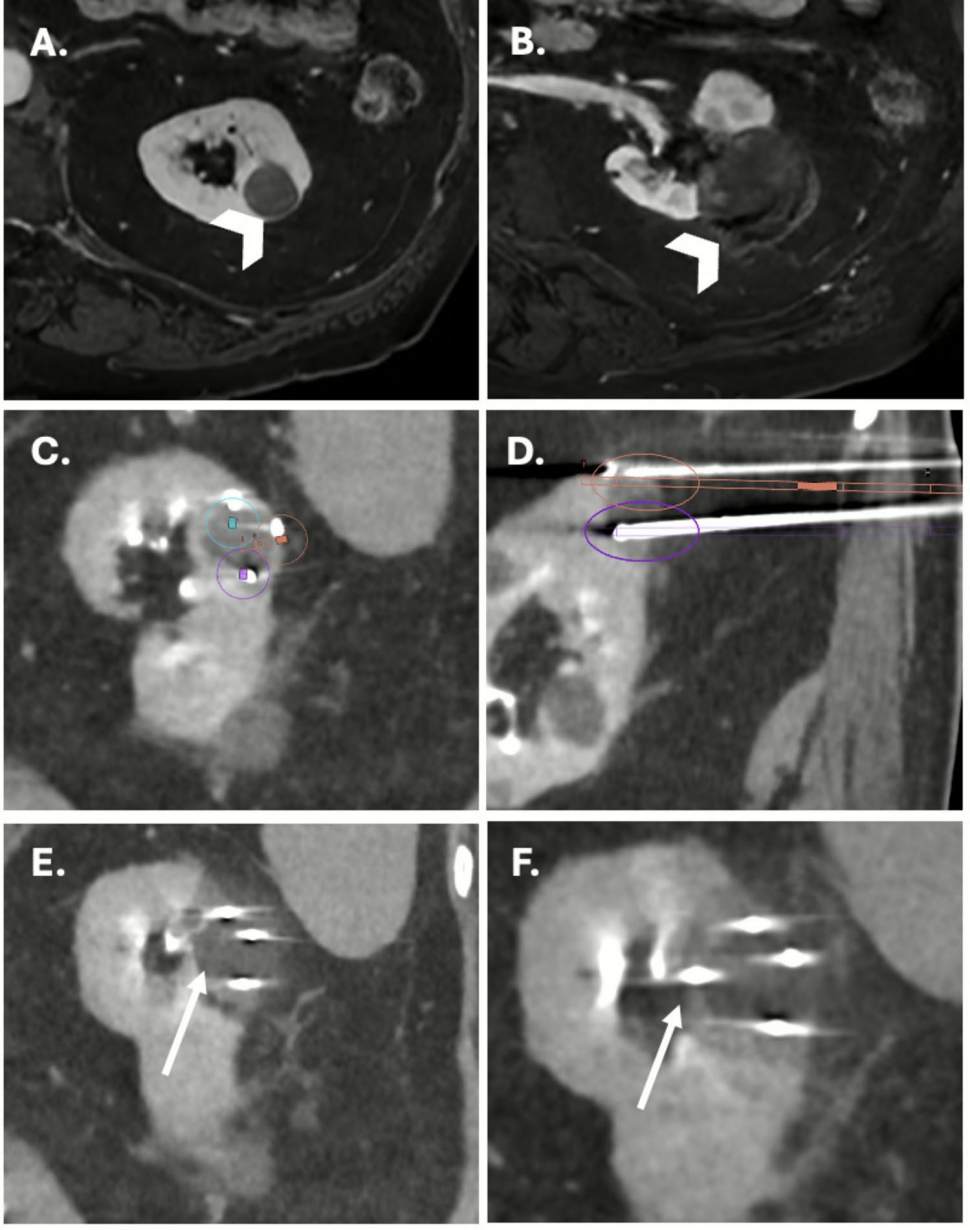
Example of robot-assisted CT-guided renal cryoablation. **A** Pre-treatment axial T1-weighted fat-saturated contrast-enhanced MRI showing a 2-cm papillary tumor of the left kidney (white arrowhead). **B** Post-treatment axial T1-weighted fat-saturated contrast-enhanced MRI demonstrating complete ablation of the lesion with no residual enhancement (white arrowhead). **C** Intraoperative coronal CT scan showing three cryoprobes inserted in bull’s eye view. The planned trajectories are overlaid and color-coded (blue, orange, violet). **D** Intraoperative sagittal CT view with two cryoprobes placed at the superior and inferior poles of the tumor. The planned trajectories are visualized as straight orange and violet lines. **E** Coronal CT image during the first freezing cycle showing the iceball adequately covering the tumor laterally and craniocaudally, but with an insufficient medial margin (white arrow). **F** Final intraoperative coronal CT image after placement of a fourth cryoprobe medially (white arrow), ensuring complete ice coverage in bull’s eye view

Robotic-assisted needle placement was technically successful in 51/54 patients, yielding a feasibility rate of 94.4% (Figs. 1, 2 and Table 2). At the needle level, 102/108 needles (94.4%) were successfully placed using robotic assistance. Non-feasibility in 3 procedures (6 needles) was attributed to robotic access limitations (possible collision with patient or table) and impaired visibility of the optical tracking system. Two occurred in the first half of the series (cases 8 and 16, 2 needles each) and one in the second half (case 53, 2 needles).

**Table 2:** Study outcomes

*Feasibility*,* n* (%)	
Per patient	51/54 (94.4)
Per needle	102/108 (94.4)
*Safety*,* n* (%)	
All adverse events	7/54 (12.9)
incl. serious adverse events	3/54 (5.6)
Accuracy*, mm, mean (± SD)	
3D deviation before adjustment	11.1 (± 7.3)
Lateral deviation before adjustment	5.3 (± 5.0)
Depth deviation before adjustment	8.8 (7.2)
3D deviation after adjustments	2.5 (± 3.7)
*Manual needle adjustments*, *n* (%)	
Depth only	54/108 (50.0)
Lateral ± depth	45/108 (41.7)
Major	6/108 (5.6)

*Calculated on all feasible needles (*n* = 102)

### Safety

During the study period, seven adverse events were reported, including three severe events. One pleural hemorrhagic effusion occurred intra-procedurally after an iatrogenic pleural puncture due to incorrect needle length entry into the software. Additionally, two post-procedural complications were reported, related to the procedure but not to the device: one abscess developed in the ablation zone, requiring drainage and re-hospitalization, and a separate case of pyelonephritis 1 month after the procedure, also requiring re-hospitalization.

### Accuracy

The needle placement accuracy before manual adjustments was 11.1 mm (± 7.3) for the 3D deviation, 5.3 mm (± 5.0) lateral deviation, and 8.8 mm (± 7.2) depth deviation (Table 2). Of 108 needles, 54 (50.0%) required a minor depth-only adjustment, 45 (41.7%) required at least a lateral adjustment (with or without depth correction), and 6 (5.6%) required a major adjustment with complete withdrawal of the needle and switch to conventional freehand insertion (Table 2). The mean 3D deviation after manual needle adjustments was 2.5 mm (± 3.7).

Subgroup analyses showed no significant difference in pre-adjustment needle placement accuracy regarding the targeted organ (liver vs. kidney), the ablation modality (microwave vs. cryoablation), the challenging status of the lesion treated (challenging vs. non-challenging), or the operator’s experience (senior vs. junior operator) (Table 3). Interestingly, post-adjustment 3D and depth accuracy were higher among junior operators (Table 3).

**Table 3:** Subgroup analyses: needle placement accuracy according to the organ-treated, the ablation modality, and the operator’s experience

	Liver	Kidney	*p*-value	MWA	Cryo	*p-value*	Challenging lesions	Non-challenging lesions	*p*-value	Seniors*	Juniors*	*p*-value
3D deviation before adjustment	11.4 (± 7.5)	9.5 (± 4.3)	*0.117*	11.7 (± 7.5)	9.9 (± 10.5)	*0.427*	11.9 (± 7.6)	10.1 (± 6.7)	*0.206*	11.7 (± 7.5)	9.9 (± 6.6)	*0.221*
Lateral deviation before adjustment	5.9 (± 5.9)	5.2 (± 3.0)	*0.446*	5.7 (± 5.5)	5.0 (± 4.7)	*0.480*	5.1 (± 5.3)	5.6 (± 4.8)	*0.605*	5.3 (± 4.5)	5.4 (± 6.1)	*0.95*
Depth deviation before adjustment	8.5 (± 7.5)	7.5 (± 4.1)	*0.552*	9.0 (± 7.7)	8.2 (± 5.8)	*0.592*	10.0 (± 7.6)	7.3 (± 6.4)	*0.057*	9.7 (± 7.5)	6.6 (± 5.9)	***0.03***
3D deviation after adjustment	2.5 (± 3.7)	2.7 (± 3.5)	*0.842*	3.0 (± 4.2)	2.1 (± 3.1)	*0.261*	11.9 (± 7.6)	11.9 (± 7.6)	*0.463*	2.7 (± 4)	1.8 (± 2.2)	***0.002***

*MWA* Microwave ablation; *Cryo* Cryoablation

*Interventional radiologists were categorized as senior if they had 5 or more years of experience; juniors: less than 5 years of experience

### Clinical outcomes

**Fig. 3:**
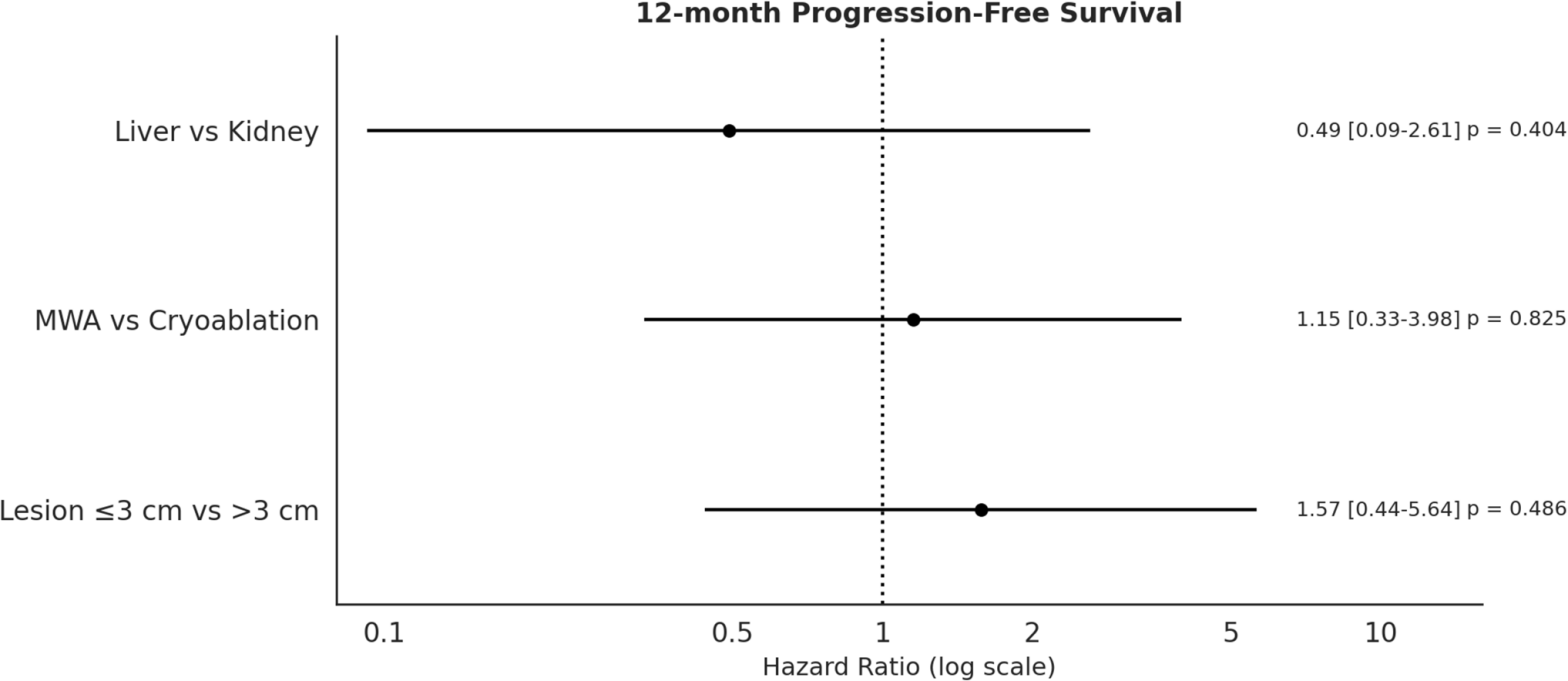
Forest plot of 12-month progression-free survival (PFS) by subgroup. The hazard ratio (HR) for progression at 12 months is shown for each subgroup comparison, along with 95% confidence intervals and *p*-values. The vertical dotted line at HR = 1.0 indicates no difference in risk between groups. No statistically significant difference in the 12-month PFS was observed according to the organ treated (liver vs. kidney), ablation modality (MWA vs. cryoablation), or lesion size (≤ 3 cm vs. > 3 cm), although point estimates suggested a trend towards improved PFS for smaller lesions and kidney targets.

Immediate ablation success was achieved in 60/61 lesions (98.4%). Ablation was not performed on one lesion due to high-risk proximity to the colon and inability to perform safe hydrodissection. The mean minimal ablation margin was 5.6 mm (± 3.2). Local tumor recurrence occurred in 4 patients (8.3%) at 2 months and in 10 patients (25.6%) at 1 year. Recurrence rates did not significantly differ by lesion location (liver vs. kidney), ablation modality (MWA vs. cryoablation), or lesion size (≤ 3 cm vs. > 3 cm) (Fig. 3).

### Operator’s satisfaction

The mean operator’s satisfaction was 3.1/4. In 48/54 procedures (88.9%), the operators rated the experience as satisfactory or very satisfactory.

## Discussion

This prospective post-market study confirms the feasibility, safety, and accuracy of robotic-assisted percutaneous thermal ablation for abdominal lesions, including liver and kidney tumors. It represents the first prospective, multi-organ evaluation of the Epione® robotic device in a real-world clinical setting.

The overall feasibility rate was high (94.4%), with 102 out of 108 needles successfully inserted using robotic guidance. The main causes of non-feasibility were accessibility issues or limited instrument visibility through the robotic device camera. Notably, most failures occurred early in the series (patients 8 and 16), during the operators’ learning curve, or in a case involving a less experienced user (patient 53). These findings and recent procedural refinements suggest that similar issues are now largely preventable in routine practice. These results are consistent with the feasibility rate previously published in the first experience of this robotic device use [17].

Several procedural optimizations have been implemented since the study inception: (i) Pre-procedural trajectory planning: Early planning before patient and device setup now enables optimized patient positioning (e.g., prone, supine, lateral, or elevated using cushions) and ensures optimal placement of the tracking reference marker, positioned between the robotic arm and the camera, reducing instrument visibility issues; (ii) Robotic arm positioning: Choosing the side of the robotic setup (right or left side of the patient) and adjusting the robotic arm angulation minimize potential collisions between the robotic arm and the patient, also reducing accessibility issues.

Importantly, no technical failures were due to intrinsic device dysfunctions; all were related to procedural workflow or device limitations that could be overcome by the user’s experience.

Safety outcomes in this study mirrored those reported for conventional freehand thermal ablation techniques [6, 23–25]. Adverse events were typical of percutaneous ablation procedures, including thermal injury to adjacent organs and hemorrhage at the puncture site. Only one complication was directly linked to the use of the robotic system—a pleural puncture resulting from incorrect needle length entry into the software, highlighting the importance of meticulous user input.

Accuracy analyses provided robust 3D, lateral, and depth deviation data before and after manual adjustments. These measurements are rarely standardized in the literature, making comparisons across studies or with other medical devices challenging if not irrelevant [26, 27]. Nevertheless, our findings fall within reported ranges. The lateral deviation, which is particularly clinically relevant, as it is harder to correct post-insertion than depth-only errors, is in range, for example, with the studies by Heerink et al*.* and Lachenmayer et al*.* [27, 28]. Of note, the accuracy was similar in challenging and non-challenging lesions, supporting previous results [17] and the idea that robotic guidance enables standardization of the technique, regardless of the difficulty of the procedure.

Most insertions required manual adjustments after the initial robotic placement, mainly limited to depth-only corrections. It is important to note that the literature on this topic remains difficult to interpret and, therefore, challenging to compare with our results. Many studies attempted to compare adjustments required during freehand procedures to those needed after robotic needle placement [29, 30]. In our opinion, this comparison lacks relevance. In freehand procedures, needles are advanced step-by-step under direct imaging guidance (CT or ultrasound) until the desired position is reached. This technique does not involve "adjustments" in the strict sense but rather represents a continuous image-guided advancement. As such, this process cannot be meaningfully compared to potential corrections made after a single, complete robotic insertion.

Theoretically, some patients’ or procedure parameters could impact needle placement accuracy, such as the thermal ablation modality (type, sharpness, thickness of the needles), the organtreated, or the operator’s experience. None has shown a significant impact on accuracy before adjustment in this study. Interestingly, only the operator’s experience impacted accuracy on the 3D deviation after manual adjustments: junior operators achieved smaller post-adjustment 3D deviations. This may reflect their greater caution and insistence on optimal alignment to try to achieve « perfectly centered» needle positioning, whereas senior operators—more accustomed to the flexibility of manual approaches—may tolerate slightly greater deviation while still achieving complete treatment.

Similarly, device upgrades have been released since the end of the study, which may impact on accuracy. Both hardware and software updates now allow enhanced avoidance of at-risk structures using the “axial mode”, which allows the rotation of the robotic arm around the axis of the needle guide. Also, reduction of the distance between the robotic arm and the skin to minimize the length of the needle trajectory in air may affect accuracy with reduced potential needle deviation or bending before skin entry. Future developments, such as a dual-sided needle guide, are also expected to further increase the flexibility in complex scenarios.

Local recurrence rates in this study, while remaining within the range reported in the literature, appear to be at the higher end, or slightly above recently published benchmarks. Several factors may explain this observation. First, the relatively large size of the treated lesions (mean diameter of 25 mm) may have contributed to increasing the recurrence risk. Second, a non negligible number of patients were lost to follow-up and subsequently excluded from the recurrence analysis, which may have artificially inflated the calculated recurrence rate. Lastly, the nature of the treated population likely played a role: several patients in our cohort had secondary neuroendocrine metastases in the setting of disseminated, slowly progressive disease. In these long-term survivors, the ablation procedures were primarily aimed at local disease control rather than complete tumor eradication, and smaller safety margins were often deliberately accepted to avoid complications and preserve organ function. These factors collectively may account for the recurrence rates observed in this cohort.

Subgroup analyses did not reveal a significant difference in the local recurrence rate depending on the organ-treated, ablation treatment delivered, or lesion size (≤ 3 cm vs. > 3 cm). However, these analyses are underpowered due to limited subgroup sample sizes, and definitive conclusions should be drawn with caution. The operator’s satisfaction was notably high, with a mean score of 3.1/4 and 88.9% of procedures rated as satisfactory or highly satisfactory. Feedback emphasized improved procedural control and precision, especially in complex cases.

This study has several limitations. First, it was conducted at a single center, with a relatively small sample size. Second, there was no control group using a conventional freehand ablation technique, which would have provided direct comparative insights. Third, the potential influence of the operators’ learning curve, particularly in early cases, may have affected outcomes. Finally, patients were selected based on an a priori decision to use robotic assistance, introducing possible selection bias.

## Conclusion

Robotic-assisted CT-guided thermal ablation of liver and kidney lesions demonstrated high feasibility, a favorable safety profile, and high needle placement accuracy in a prospective post-market setting. These results support the reliability of robotic guidance for percutaneous abdominal interventions, including in anatomically complex cases. Further comparative and larger-scale studies are warranted to better define its impact on clinical outcomes and procedural efficiency.

## Supplementary Information

Below is the link to the electronic supplementary material.

Supplementary Video 1. Trajectory planning for robotic-assisted microwave ablation of a hepatic dome lesion. Screen capture of the robotic planning software showing CT-based trajectory planning for microwave ablation of a hepatic dome lesion, including definition of the skin entry point and target location.
Supplementary file1 (MP4 68595 KB)

Supplementary Video 2. Robotic-assisted needle insertion for microwave ablation of a liver lesion. Intra-procedural video illustrating robotic-assisted insertion of a microwave ablation needle into the liver along the pre-planned trajectory during a CT-guided intervention.
Supplementary file2 (M4V 26449 KB)
